# Mental wellbeing among Hispanic female domestic cleaners

**DOI:** 10.1186/s13690-020-0390-9

**Published:** 2020-02-20

**Authors:** Jennifer Ish, David Gimeno Ruiz de Porras, Kristina W. Whitworth

**Affiliations:** 1 Southwest Center for Occupational and Environmental Health (SWCOEH), Department of Epidemiology, Human Genetics & Environmental Sciences, School of Public Health in San Antonio, The University of Texas Health Science Center at Houston (UTHealth), 7411 John Smith Drive, Suite 1100, San Antonio, TX 78229 USA; 2 Center for Research in Occupational Health (CiSAL), Universitat Pompeu Fabra, Barcelona, Catalonia Spain; 3 CIBER of Epidemiology and Public Health, Barcelona, Spain

**Keywords:** WHO-5, Mental health, Informal employment, Psychosocial stressors, Worker wellbeing

## Abstract

**Background:**

The purpose of this study was to examine the mental wellbeing of self-employed, Hispanic female domestic cleaners in San Antonio, Texas.

**Methods:**

We conducted a cross-sectional pilot study and administered a short questionnaire to 56 participants. Mental wellbeing was assessed using The World Health Organization Well-Being Index (WHO-5). We calculated the age-adjusted prevalence of poor mental wellbeing, both overall and stratified by socioeconomic, neighborhood, and health characteristics.

**Results:**

Almost half of the participants screened positive for poor mental wellbeing (47.3%) with a mean WHO-5 score of 68.9 [standard error (SE) = 3.1]. We observed a high prevalence of poor mental wellbeing among participants with less than a high school education (56.0%), who worked less than 30 h per week (57.0%) and who sometimes or always felt unsafe at her cleaning job (69.1%).

**Conclusions:**

Female domestic cleaners, particularly those who work in the informal sector, are an overburdened and understudied population. This is particularly true regarding their mental wellbeing, which has largely been considered as an afterthought in epidemiologic studies of cleaning workers in general. Our results suggest that this group of domestic cleaners faces several psychosocial stressors, both in and outside of the workplace, and may have a high risk of poor mental health outcomes.

## Background

Workers in the cleaning industry are confronted with many workplace health risks, such as musculoskeletal injury and chemical hazards [1, 2]. However, little attention has been given to psychosocial risk factors that negatively affect cleaners’ mental health. Further, there is little to no information on mental health among United States (US)-based domestic cleaners; most research has been conducted among industrial or commercial cleaners and/or domestic cleaners outside of the US, even though nearly one million workers in the US were employed as maids or housekeeping cleaners in 2017 [3].

Cleaners face a multitude of workplace stressors, including low job control, unpredictable working hours, job insecurity, demanding workloads, lack of career development, and isolation [4]. These work-related psychosocial stressors have been reported among hotel cleaners in the US [5, 6] and immigrant female household cleaners and caregivers in Spain [7]. Having a stressful working environment can in turn negatively impact mental health [8]. In Norway, Gamperiene et al. [9] found a greater burden of mental health problems among female industrial cleaners compared to the general female Norwegian population. To our knowledge, only one study has examined mental health specifically among domestic cleaners. Results from a cross-sectional analysis of 335 women in Salvador, Brazil, indicates that being employed as a housemaid is positively associated with anxiety symptoms [10].

Domestic cleaners are a particularly vulnerable group, with poor legal and social protections [11]. This group is generally excluded from US labor laws, and those who are self-employed often work without the protection of formal employment contracts. This may create precarious employment conditions that compound workplace psychosocial stressors [12, 13]. Further, domestic cleaners also confront unique challenges given their socioeconomic and, in some cases, immigration status. In Texas, women who work as domestic cleaners may be further disadvantaged as they are primarily low-income, Hispanic, and may be undocumented. Economic and social deprivation, together with precarious employment conditions and workplace psychosocial stressors, may adversely impact the mental health of women working as domestic cleaners.

Understanding the mental health of US-based domestic cleaners is important to addressing their occupational health and safety in a holistic manner, particularly in the broader context of the Total Worker Health® framework [14]. To this end, the objective of the present study is to characterize the prevalence of poor mental wellbeing among self-employed, female domestic cleaners in San Antonio, Texas.

## Methods

A cross-sectional pilot study was conducted among women working as domestic cleaners in San Antonio, Texas in 2017 [15]. The goal of this pilot study was to assess the feasibility of recruiting a hard-to-reach and vulnerable population of Hispanic women working as domestic cleaners and assess the prevalence of various occupational and environmental hazards and health status of this population. We recruited women to this study in collaboration with Domésticas Unidas (DU), a local San Antonio-based grassroots organization affiliated with the National Domestic Workers Alliance. Women were recruited at monthly DU meetings which were open to all domestic workers (including house cleaners, home health aides, nannies, etc.) in the San Antonio area, regardless of whether they are DU members. Almost all individuals attending these meetings are women. DU utilizes these monthly meetings for a variety of purposes, for example, to update the community on their advocacy work and offer training focused on developing job skills, negotiating salary and time off, and safe use of cleaning products. At these meetings, DU also provides women with information and tools to protect their rights on the job as well as the opportunity to share their experiences. Also, we recruited women for our study at community events such as health fairs. At all these venues, Spanish-speaking study staff (DGRP and graduate research assistants trained in responsible conduct of research and data collection) approached women to explain the purpose of the research and ask if they would like to participate. Women were eligible if they had worked as self-employed house cleaner in the past 12 months. A total of 56 women were included in the pilot study and administered a short survey in Spanish. Before administering the survey, study staff provided participants with an information page that assured participants of the voluntary nature of their participation and that their responses would remain confidential. Additionally, all participants were informed of their ability to withhold responses, terminate participation, and provided staff contact information should they have questions or concerns about the study. By completing the survey, participants granted implied consent to participate in the research. The study protocol was approved by The University of Texas Health Science Center at Houston (UTHealth) Committee for the Protection of Human Subjects. Women were asked to self-report demographic information, smoking status, work history, and several chronic health conditions, including whether they have ever had high blood pressure. Musculoskeletal pain was assessed by asking participants if they have had trouble (ache, pain, discomfort) in their neck, shoulders, elbows, wrists/hands, upper back, lower back, hips/thighs, knees, or ankles/feet in the past 12 months [16]. The self-reported World Health Organization Well-Being Index questionnaire (WHO-5) was used to assess mental wellbeing [17]. The WHO-5 asks participants to report to what extent they felt cheerful and in good spirits, calm and relaxed, active and vigorous, refreshed and well rested, and that their daily activities had been interesting to them, in the previous two-weeks. Responses included: “at no time”, “some of the time”, “less than half of the time”, “more than half of the time”, “most of the time”, or “all of the time” and were coded as 0 (representing worst possible quality of life) to 5 (representing best possible quality of life). A raw score was calculated as the sum of the five items. The total raw scores can range from 0 (i.e., worst possible mental wellbeing) to 25 (best possible). As recommended, a score below 13 or a response of ‘at no time’ or ‘some of the time’ to any of the items represents poor mental wellbeing [17]. Additionally, a summary percentage score was calculated by multiplying the total raw scores by 4. The summary percentage scores range from 0 (i.e., worst possible wellbeing) to 100 (best possible). Other survey details have been reported elsewhere [15].

We calculated the age-adjusted prevalence of poor mental wellbeing, both overall and stratified by socioeconomic, neighborhood, and health characteristics. All analyses were performed using SAS version 9.4 (Cary, North Carolina).

## Results

More than half of the women in the study (57.1%) were over 50 years of age. The youngest participant was 23 and the oldest was 74 years of age. Almost all (96.4%) women identified as Hispanic, 57.1% had an annual household income at or below $15,000, and 71.4% had a high school education or less. The number of years lived in the US ranged from 1 to 70 while the median was 24.5 years. Only four women (7.6%) were current smokers. Half of the participants (50.0%) worked a total of 30 or more hours per week, although only 28.6% reported working all of these hours as a cleaner.

Almost half of the participants screened positive for poor mental wellbeing (47.3%) with a mean WHO-5 score of 68.9 (95% CI: 62.6–75.2) (see Table 1). The highest prevalence of poor mental wellbeing was among women with less than an high school education (56.0%), who sought medical care at a private doctor’s office (63.9%), worked less than 30 h per week as a cleaner (57.0%), lived alone (58.4%), and who sometimes or always felt unsafe at her cleaning job (69.1%). Among women who reported having musculoskeletal pain, over half (55.5%) screened positive for poor mental wellbeing, compared to only 17.3% of women with no musculoskeletal pain. There was a slightly higher prevalence of poor mental wellbeing among women who reported having high blood pressure and symptoms of bronchial hyper-responsiveness than women who did not have these conditions (51.2% vs. 44.0 and 55.8% vs. 43.1%, respectively). None of the differences in the prevalence of poor mental wellbeing between groups were statistically significant.

**Table 1:** Mental wellbeing among 56 Hispanic female domestic cleaners, San Antonio, Texas, 2017

	n (%)		Age-adjusted prevalence (%) of poor mental wellbeing, mean (SE) ^A^		WHO-5 score, mean (SE) ^A^	
Overall	56	(100)	47.3	(6.5)	68.9	(3.1)
Education						
Less than High School	20	(35.7)	56.0	(10.9)	67.1	(5.3)
High School	20	(35.7)	46.6	(10.8)	71.7	(5.1)
College or higher	16	(28.6)	37.3	(12.3)	67.0	(6.0)
Annual household income ($)^B^						
≤ 15,000	32	(57.1)	49.2	(8.8)	66.7	(4.1)
15,001+	23	(41.1)	43.4	(10.1)	72.0	(4.7)
Place most often sought for medical care^B^						
Private doctor’s office	11	(19.6)	63.9	(13.6)	61.1	(7.1)
Public clinic or ER	42	(75.0)	43.6	(7.4)	70.0	(3.5)
Avg. hours/week worked as cleaner						
≤ 29	40	(71.4)	57.0	(7.5)	66.2	(3.7)
30+	16	(28.6)	25.8	(10.2)	74.9	(5.8)
Live alone						
Yes	10	(17.9)	58.4	(15.0)	66.5	(7.5)
No	46	(82.1)	45.1	(7.2)	69.3	(3.4)
Feel unsafe at cleaning job^B^						
Never	48	(87.3)	43.2	(7.1)	70.7	(3.3)
Sometimes or always	7	(12.7)	69.1	(17.6)	59.1	(8.5)
Feel unsafe in neighborhood						
Never	32	(57.1)	46.1	(8.8)	71.7	(4.1)
Sometimes or always	24	(42.9)	49.5	(10.2)	65.0	(4.7)
Think that crime/violence is a problem in neighborhood^B^						
No	39	(70.9)	44.7	(7.9)	70.2	(3.7)
Yes	16	(29.1)	50.9	(12.5)	65.9	(5.8)
High blood pressure						
No	34	(63.0)	44.0	(8.7)	71.1	(4.2)
Yes	20	37.0)	51.2	(12.2)	65.1	(5.9)
Musculoskeletal pain^D^						
No	12	(21.8)	17.3	(11.3)	83.0	(6.5)
Yes	43	(78.2)	55.5	(7.0)	64.9	(3.3)
Symptoms of bronchial hyper-responsiveness^E^						
No	36	(65.5)	43.1	(8.1)	71.9	(3.7)
Yes	19	(34.5)	55.8	(11.2)	62.9	(5.2)

*n (%)* frequency (percentage); *SE* standard error; *WHO-5* World Health Organization Well-being Index

^A^ None of the differences are statistically significant

^B^ *n* = 1 missing

^C^ *n* = 3 missing

^D^ self-reported trouble (ache, pain, discomfort) in neck, shoulders, elbows, wrists/hands, upper back, lower back, hips/thighs, knees, or ankles/feet in the past 12 months; *n* = 2 missing

^E^ Symptoms of bronchial hyper-responsiveness assessed by an 8-item questionnaire related to respiratory symptoms in the past 12 months [18], *n* = 2 missing

## Discussion

Our pilot study provides data informing the prevalence of poor mental wellbeing among a group of informal domestic cleaners in San Antonio. The WHO-5, which was used to assess mental wellbeing in this study, has been validated as a screening tool for depression in many populations [17]. We did not intend to use the questionnaire to screen for depression and cannot directly compare our results to other depression prevalence estimates. Nonetheless, the high proportion of women in our study who met the positive screening criteria signals that they may have a large burden of mental health problems. To our knowledge, the WHO-5 index has not been used among domestic cleaners; however, the WHO-5 scores of the women in this study is similar to that of informal caregivers in the 2016–2017 European Quality of Life Survey who had a mean score of 60.84 [19].

Women with more favorable socioeconomic indicators (i.e., higher household incomes and educational attainment) had a lower prevalence of poor mental wellbeing than women with less favorable socioeconomic indicators. This could reflect well-documented socioeconomic disparities in mental health [20]. Additionally, women who reported working less than 30 h per week had higher prevalence of poor mental health than women who reported working more hours. While many of these women were working another job, perhaps they faced job insecurity and financial stress. This could indicate that these women faced increased employment precariousness, an important social determinant of health [12]. Also, more women who were concerned with safety at their jobs had poor mental wellbeing than those who never felt unsafe. This pattern was similar but attenuated for women who felt unsafe in their neighborhood or felt that violence and/or crime was a problem where they lived. Feeling unsafe may indicate that these women have experienced street victimization, violence at work, or even intimate partner violence, all of which are risk factors for negative health behaviors, chronic disease, and mental health problems [21].

It is unclear why, in this sample, women who sought medical care at a private doctor’s office had a higher prevalence of poor mental health than those who sought care elsewhere, but it could be indicator of severity such that women with known mental health problems or illness seek specialized care. Our observation of a higher prevalence of poor mental well-being among women who report chronic health conditions (e.g., musculoskeletal pain) is not surprising given well-documented associations between mental health and chronic illness [22]. None of the observed differences in mental wellbeing were statistically significant, perhaps due to the small sample size of the study. Nevertheless, the observations of this study suggest that the mental health of this group of informal domestic cleaners may be affected by socioeconomic, job and neighborhood factors.

Given the nature of this pilot study, these analyses were limited by a small sample size and limited power. Second, given that the participants were recruited from one local organization, our sample may not be representative of all informal, Hispanic domestic cleaners in the San Antonio area. Lastly, there is potential for recall bias and thus, error in our estimates of the prevalence of mental wellbeing.

## Conclusions

These data suggest that this group of domestic cleaners faces several psychosocial stressors, both in and outside of the workplace, and may have a high risk of poor mental health outcomes. To date, the majority of research on cleaners and mental health has been conducted outside the US and/or among industrial cleaners; thus, very little data exist regarding this particularly vulnerable group of cleaners in the US. Despite the cross-sectional pilot nature of our study, it nonetheless provides data to begin to fill in this gap. More research is needed to understand what factors contribute to poor mental health outcomes among domestic cleaners. Further, a holistic approach, such as that offered by the Total Worker Health framework, can aid efforts to improve working conditions and protections for domestic cleaners as well as enhance their health and quality of life [14].

## Data Availability

The dataset analyzed during the current study is available from the corresponding author on reasonable request.

## Abbreviations

DU: Domésticas Unidas
WHO-5: The World Health Organization Well-Being Index

## References

[1] Zock JP (2005). World at work: cleaners. Occup Environ Med.

[2] Arif AA, Hughes PC, Delclos GL (2008). Occupational exposures among domestic and industrial professional cleaners. Occup Med.

[3] U.S. Bureau of Labor Statistics (2017). Current Population Survey.

[4] European Agency for Safety and Health at Work (2009). The occupational safety and health of cleaning workers.

[5] Hsieh YC, Apostolopoulos Y, Sonmez S (2013). The world at work: hotel cleaners. Occup Environ Med.

[6] Hsieh Y, Apostolopoulos Y, Sönmez S (2016). Work conditions and health and well-being of Latina hotel housekeepers. J Immigr Minor Health.

[7] Ahonen EQ, López-Jacob MJ, Vázquez ML, Porthé V, Gil-González D, García AM (2010). Invisible work, unseen hazards: the health of women immigrant household service workers in Spain. Am J Ind Med.

[8] Leka S, Jain A, World Health Organization (2010). Health impact of psychosocial hazards at work: an overview.

[9] Gamperiene M, Nygård JF, Sandanger I, Wærsted M, Bruusgaard D (2006). The impact of psychosocial and organizational working conditions on the mental health of female cleaning personnel in Norway. J Occup Med Toxicol.

[10] Sales EC, Santana VS (2003). Depressive and anxiety symptoms among housemaids. Am J Ind Med.

[11] Burnham L, Theodore N (2012). Home economics: the invisible and unregulated world of domestic work.

[12] Vives A, Amable M, Ferrer M, Moncada S, Llorens C, Muntaner C (2013). Employment precariousness and poor mental health: evidence from Spain on a new social determinant of health. J Environ Public Health.

[13] Han K, Chang J, Won E, Lee M, Ham B (2017). Precarious employment associated with depressive symptoms and suicidal ideation in adult wage workers. J Affect Disord.

[14] Chari R, Chang C, Sauter SL, Sayers ELP, Cerully JL, Schulte P (2018). Expanding the paradigm of occupational safety and health: a new framework for worker well-being. J Occup Environ Med.

[15] Whitworth KW, Berumen-Flucker B, Delclos GL, Fragoso S, Mata C, Gimeno Ruiz de Porras D. Job hazards and respiratory symptoms in Hispanic female domestic cleaners. Arch Environ Occup Health. 2019;1–5. [Online]. Available from: http://search.proquest.com/docview/2216774543/. 10.1080/19338244.2019.1606774 PMC8849900 31033410

[16] Kuorinka I, Jonsson B, Kilbom A, Vinterberg H, Biering-Sørensen F, Andersson G (1987). Standardised Nordic questionnaires for the analysis of musculoskeletal symptoms. Appl Ergon.

[17] Topp CW, Østergaard SD, Søndergaard S, Bech P (2015). The WHO-5 well-being index: a systematic review of the literature. Psychother Psychosom.

[18] Delclos GL, Gimeno D, Arif AA, Burau KD, Lusk C, Stock T (2007). Occupational risk factors and asthma among health care professionals. Am J Respir Crit Care Med.

[19] Maguire Rebecca, Hanly Paul, Maguire Phil (2019). Beyond care burden: associations between positive psychological appraisals and well-being among informal caregivers in Europe. Quality of Life Research.

[20] Lorant V, Deliège D, Eaton W, Robert A, Philippot P, Ansseau M (2003). Socioeconomic inequalities in depression: a meta-analysis. Am J Epidemiol.

[21] Heise L, Ellsberg M, Gottmoeller M (2002). A global overview of gender-based violence. Int J Gynecol Obstet.

[22] Fortin M, Lapointe L, Hudon C, Vanasse A, Ntetu AL, Maltais D (2004). Multimorbidity and quality of life in primary care: a systematic review. Health Qual Life Outcomes.

